# Repeated use of SSRIs potentially associated with an increase on serum CK and CK-MB in patients with major depressive disorder: a retrospective study

**DOI:** 10.1038/s41598-021-92807-7

**Published:** 2021-06-28

**Authors:** Shengwei Wu, Yufang Zhou, Zhengzheng Xuan, Linghui Xiong, Xinyu Ge, Junrong Ye, Yun Liu, Lexin Yuan, Yan Xu, Guoan Ding, Aixiang Xiao, Jianxiong Guo, Lin Yu

**Affiliations:** 1 Affiliated Brain Hospital of Guangzhou Medical University (Guangzhou Huiai Hospital), No. 36, Mingxin Road, Liwan District, Guangzhou, 510370 Guangdong China; 2 Guangdong Provincial Hospital of Traditional Chinese Medicine, Guangzhou, 510120 China; 3 Guangzhou University of Chinese Medicine, Guangzhou, 510006 China

**Keywords:** Drug safety, Depression

## Abstract

There is a large amount of evidence that selective serotonin reuptake inhibitors (SSRIs) are related to cardiovascular toxicity, which has aroused concern regarding their safety. However, few studies have evaluated the effects of SSRIs on cardiac injury biomarkers, such as creatine kinase (CK) and creatine kinase isoenzyme (CK-MB). The purpose of our study was to determine whether SSRIs elevated CK and CK-MB levels of prior medicated depressive patients (PMDP) compared to first-episode drug-naïve depressive patients (FDDPs). We performed an observational and retrospective study involving 128 patients with major depressive disorder. Patients who had never used any type of antidepressant were designated FDDP; patients who had used only one type of SSRI but were not treated after a recent relapse were designated PMDP. Serum CK and CK-MB levels were measured before and after using SSRIs for a period of time. The duration of current treatment in the FDDP and PMDP groups was 16.200 ± 16.726 weeks and 15.618 ± 16.902 weeks, respectively. After SSRI treatment, levels of serum CK in the PMDP group were significantly higher than in the FDDP group. Univariate ANCOVA results revealed that PMDP was 22.313 times more likely to elevate CK (*OR* 22.313, 95% *CI* 9.605–35.022) and 2.615 times more likely to elevate CK-MB (*OR* 2.615, 95% *CI* 1.287–3.943) than FDDP. Multivariate ANCOVA revealed an interaction between the group and sex of CK and CK-MB. Further pairwise analysis of the interaction results showed that in female patients, the mean difference (*MD*) of CK and CK-MB in PMDP was significantly greater than that in FDDP (*MD* = 33.410, *P* = 0.000, 95% *CI* 15.935–50.886; *MD* = 4.613, *P* = 0.000, 95% *CI* 2.846–6.381). Our findings suggest that patients, especially females, who had previously used SSRI antidepressants were more likely to have elevated CK and CK-MB, indicators of myocardial muscle injury. Use of SSRIs should not be assumed to be completely safe and without any cardiovascular risks.

## Introduction

Due to tremendous pressure brought on by unprecedented economic development and social change, the incidence rates of mental disorders have dramatically increased. Depression is one of the most common mental disorders and is listed as having the second largest disease burden in the world. A recent epidemiological survey in China reported that the weighted lifetime prevalence of mental disorders in adults was 16.6%, in which the prevalence of anxiety disorders, mood disorders (primarily depressive disorder) and substance abuse disorders was 7.6%, 7.4% and 4.7%, respectively^1^. SSRIs are the most commonly prescribed antidepressants in China, but their long-term health effects are still controversial. They are considered to be safer and more suitable for patients with cardiovascular disease (CVD) than traditional tricyclic antidepressants (TCAs)^2,3^. However, there is growing evidence that SSRIs are also associated with cardiovascular toxicity, such as arrhythmias, prolonged QTc intervals^4^ and orthostatic hypotension^5^. Studies have shown that the use of SSRIs is associated with an increased risk of major adverse cardiovascular events, including sudden death^6,7^. In view of the risk of prolonged QTc, the US Food and Drug Administration (FDA) published a drug security warning in 2011, which declared that Citalopram, an SSRI, extends the QTc interval in a dose-dependent manner^8^. However, Kahl et al. reported no significant correlation between SSRIs and QTc interphase^9^. Nevertheless, whether SSRIs are associated with an elevated risk of cardiovascular events remains uncertain.

Creatine kinase (CK) is one enzyme that catalyses the reversible phosphorylation of adenosine triphosphate (ATP) and creatine to adenosine diphosphate (ADP) and phosphocreatine, which is primarily distributed in bone and myocardium. The plasma concentration of creatine kinase isoenzyme (CK-MB), one of the isoenzymes of CK, is generally used to evaluate acute coronary syndrome. The detection of serum CK isozymes, especially the mass concentration of serum CK-MB, is helpful for judging the degree of myocardial injury. Few studies have investigated the relationship between SSRIs and CK or CK-MB, although animal experiments have shown that high doses of sertraline may cause cardiotoxic effects^10^. There is some new evidence that SSRIs may have some effects on foetal cardiovascular cells during foetal development, both in vitro and in vivo. Specifically, in vitro studies have observed that SSRIs, including fluoxetine and sertraline, slow calcium oscillations in cardiac myocytes and increase cardiac injury biomarkers, including CK-MB^11^. However, it is not clear whether SSRIs have an effect on serum CK and CK-MB in patients with depression.

The purpose of our study was to examine whether SSRIs elevated CK and CK-MB levels in prior medicated depressive patients (PMDP) compared with drug-naïve depressive patients (FDDP). To our knowledge, this is the first study to investigate the effects of SSRIs on serum CK and CK-MB levels in patients with major depressive disorder (MDD).

## Methods

### Patients

This was an observational, retrospective study that obtained informed consent from all subjects, and this research was approved by the Ethics Committee of the Affiliated Brain Hospital of Guangzhou Medical University (Guangzhou Huiai Hospital) with code number (2017) NO.003. The study followed the ethical principles of the Declaration of Helsinki 1964.

Subjects in this study were patients with MDD who were treated in the outpatient clinic of the Affiliated Brain Hospital of Guangzhou Medical University (Guangzhou Huiai Hospital) from January 2018 to December 2018. The following inclusion criteria were applied for subjects: (a) Han Chinese, (b) 15–66 years old, (c) a diagnosis of unipolar depression in accordance with the fifth edition of the Diagnostic and Statistical Manual of Mental Disorders (DSM-V), and (d) patients with MDD who had never been treated with any type of antidepressant or SSRI drugs (if they had, they had only used one SSRI, but had not received treatment after a recent relapse).

We excluded participants who were diagnosed with CVD, chronic heart failure, arrhythmia or other related complications, as these diseases could influence outcomes. Participants with any disease known to affect the activity of serum CK and CK-MB^12,13^ (i.e., acute psychosis, seizure, acute cerebrovascular disease, hyperthyroidism, malignancy, renal failure, muscle disease, physical agitation, substance use disorder within the preceding three months) were also excluded from the study. Since antidepressant polypharmacy might increase the risk of adverse effects, participants who were prescribed more than one antidepressant or who were concurrently prescribed mood stabilizers or other psychotropic substances were excluded.

SSRIs included citalopram, sertraline, fluvoxamine, fluoxetine and paroxetine. After diagnosis by participating psychiatrists, participants were prescribed one of the SSRIs mentioned above based on clinical evidence and the standard dose range of antidepressants. Both FDDP and PMDP received the same medications after onset. Finally, citalopram was used in 47 cases, and sertraline, fluvoxamine, fluoxetine and paroxetine were used in 34, 21, 15 cases and 11 cases, respectively. Participants were asked not to consume any alcohol or to exercise excessively during the study.

### Demographic information

We only collected data on sex, age, depression duration, duration of current treatment and medication status of patients limited to retrospective studies.

### Blood sample collection and storage

After overnight fasting, blood samples were collected before breakfast and medication administration. Afterward, at room temperature, blood samples were allowed to naturally coagulate for an hour, and then the serum was extracted from the blood sample by centrifugation at 1000 × *g* for 15 min. Finally, serum samples were stored at − 80 °C until further analysis.

CK levels were detected using a creatine kinase assay kits (DGKC method), and CK-MB levels were detected using a creatine kinase isoenzyme assay kits (immunoinhibition method). The manufacturer was Hunan Yonghe Sunshine Biological Technology Co., Ltd. The production date and batch number of these kits could not be traced due to retrospective nature of this study. The specific methods were recommended by the German Society of Clinical Chemistry^14^. The normal ranges of CK and CK-MB established by the above method were 26–174 U/L and 0–24 U/L, respectively.

### Statistical methods

We used descriptive and multivariable approaches. All analyses were completed using SPSS 19.0. Descriptive statistics were used to determine the baseline variable distribution of FDDP and PMDP in terms of sex, age, duration of depression, and duration of current treatment. Continuous variables were examined by parameters, and classified variables were tested by chi-square. Covariance analysis (ANCOVA) was used to compare differences between FDDP and PMDP in CK and CK-MB. First, univariate ANCOVA was used to compare all variables, and then stratified univariate ANCOVA and multivariate ANCOVA were further used for the difference results. Pairwise comparison analysis was conducted for results that demonstrated interaction. A *P* value (bilateral) less than 0.05 was considered to be statistically significant.

## Results

### General characteristics of subjects

The sociodemographic and biophysical characteristics of the study population are summarized in Table 1. There were no statistically significant differences between the two groups in terms of male/female ratio, age, or duration of current treatment. However, the disease duration in the PMDP group was significantly longer than in the FDDP (*P* < 0.05).

**Table 1:** Baseline general characteristics of FDDP and PMDP subjects.

Variables	FDDP	PMDP	*t/chi-square test*	*P*
**Sex**				
Male	24 (40.0)	38 (55.9)	3.219	0.073
Female	36 (60.0)	30 (44.1)		
**Age (year)**	34.667 ± 14.237	31.588 ± 13.125	1.273	0.206
Below 30 years	28 (46.7)	42 (61.8)	2.932	0.087
Above 30 years	32 (53.3)	26 (38.2)		
**Duration of depression (week)**	48.520 ± 35.476	169.22 ± 184.905	4.974	0.000
Within 100 weeks	46 (76.7)	32 (47.1)	11.739	0.001
More than 100 weeks	14 (23.3)	36 (52.9)		
**Duration of current treatment (week)**	16.200 ± 16.726	15.618 ± 16.902	0.195	0.845
Within 10 weeks	32 (53.3)	36 (52.9)	0.002	0.965
More than 10 weeks	28 (46.7)	32 (47.1)		

Variables are shown as N (%) and mean ± SD.

### Results of serum levels of CK and CK-MB between FDDP and PMDP

The laboratory results are shown in Table 2. At baseline, there was no difference in CK between the two groups, but levels of serum CK-MB in the PMDP group were higher than in the FDDP group (*P* = 0.001). After SSRI treatment, levels of CK and CK-MB in the PMDP group were significantly higher than in the FDDP group (*P* = 0.005, *P* = 0.000, respectively).

**Table 2:** Results of serum levels of CK and CK-MB in FDDP and PMDP groups.

	N	Creatine kinase (U/L)		Creatine kinase-MB (U/L)	
		Baseline	After treatment	Baseline	After treatment
FDDP	60	116.000 ± 34.077	120.101 ± 36.583	11.770 ± 2.738	13.830 ± 3.083
PMDP	68	114.971 ± 41.476	142.353 ± 35.810	13.982 ± 5.689	16.464 ± 4.111
*t*		− 0.152	2.854	3.473	4.129
*P*		0.879	0.005	0.001	0.000

Variables are shown as the mean ± SD.

### Covariance analysis of factors related to elevated CK and CK-MB

Using baseline CK and CK-MB as covariates and analysing the variables (group, sex, age, duration of depression, duration of current treatment) separately, potential risk factors associated with increased CK and CK-MB were determined. From Table 3, we observed that, compared to FDDP, PMDP was approximately 22.313 times more likely to have an increase in CK (*OR* 22.313, 95% *CI* 9.605–35.022). Furthermore, CK-MB in PMDP was approximately 2.615 times more likely to increase than in FDDP (*OR* 2.615, 95% *CI* 1.287–3.943). In addition to medication history, sex was also a risk factor for increased CK. Specific analysis (Table 4) revealed that after treatment, increased of CK and CK-MB in female PMDP were 38.097 and 4.591 times higher, respectively, than in female FDDP and these differences were statistically significant (*P* = 0.000, 95% *CI* 22.832–53.362; *P* = 0.000, 95% *CI* 2.774–6.408). However, the differences in CK and CK-MB between the two groups in male subjects after treatment were not statistically significant.

**Table 3:** Univariate covariance analysis of factors related to elevated CK and CK-MB.

Variables	CK							CK-MB						
	*F*	*R*^*2*^	Observed power	*P*	*OR*	*95% CI*		*F*	*R*^*2*^	Observed power	*P*	*OR*	*95% CI*	
						Lower	Upper						Lower	Upper
Group (PMDP/FDDP)	12.076	0.076	0.932	0.001	22.313	9.605	35.022	15.191	0.101	0.972	0.000	2.615	1.287	3.943
Sex	12.060	0.076	0.931	0.001	22.940	9.866	36.014	0.016	− 0.008	0.052	0.900	− 0.087	− 1.454	1.279
Age	0.022	− 0.013	0.053	0.882	1.016	− 12.477	14.508	1.754	0.006	0.260	0.188	0.910	− 0.450	2.270
Disease duration	0.796	− 0.006	0.143	0.374	6.126	− 7.466	19.718	0.908	0.000	0.157	0.343	0.676	− 0.729	2.082
Duration of current therapy	0.002	− 0.013	0.050	0.963	− 0.311	− 13.647	13.025	0.296	− 0.005	0.588	0.588	− 0.376	− 1.743	0.992

The baseline level of CK or CK-MB was taken as a covariate.

**Table 4:** Univariate covariance analysis results of elevated CK and CK-MB of PMDP compared to FDDP in male and female patients.

Variables	Male							Female						
	*F*	*R*^*2*^	Observed Power	*P*	*OR*	*95%CI*		*F*	*R*^*2*^	Observed Power	*P*	*OR*	*95%CI*	
						Lower	Upper						Lower	Upper
CK	0.390	− 0.023	0.094	0.535	6.594	− 14.545	27.732	24.872	0.261	0.998	0.000	38.097	22.832	53.362
CK-MB	0.375	− 0.027	0.093	0.543	0.587	− 1.331	2.505	25.492	0.280	0.999	0.000	4.591	2.774	6.408

The baseline level of CK or CK-MB was taken as a covariate.

According to the results of univariate ANCOVA in Table 3, multivariate ANCOVA was performed on variables with significant differences. The results showed an interaction between the group and sex of CK and CK-MB (all *P* < 0.05) (Table 5). Further pairwise analysis of the interaction results demonstrated that in female patients, the mean difference (*MD*) of CK and CK-MB in PMDP was significantly greater than in FDDP, respectively (*MD* = 33.410, *P* = 0.000, 95% *CI* 15.935–50.886; *MD* = 4.613, *P* = 0.000, 95% *CI* 2.846–6.381), while there were no significant difference in the *MD* of CK and CK-MB between the two groups in male patients (Table 6).

**Table 5:** Multivariate covariance analysis of factors related to elevated CK and CK-MB.

Variables	CK						CK-MB					
	*F*	Observed power	*P*	*OR*	*95% CI*		*F*	Observed power	*P*	*OR*	*95% CI*	
					Lower	Upper					Lower	Upper
Group (PMDP/FDDP)	8.953	0.843	0.003	33.410	15.935	50.886	15.428	0.974	0.000	4.613	2.846	6.381
Sex	9.458	0.862	0.003	34.462	16.417	52.506	0.252	0.079	0.617	1.712	− 0.145	3.568
Group*Sex	5.174	0.617	0.025	–	–	–	10.109	0.884	0.002	–	–	–

The baseline level of CK or CK-MB was taken as a covariate.

**Table 6:** Pairwise analysis of interactions.

Sex	CK							CK-MB						
	*(I)*	*(J)*	*MD (I-J)*	*SE*	*P*	*95% CI*		*(I)*	*(J)*	*MD (I-J)*	*SE*	*P*	*95% CI*	
						Lower	Upper						Lower	Upper
Male	PMDP	FDDP	3.755	9.175	0.683	− 14.407	21.916	PMDP	FDDP	0.548	0.940	0.561	− 1.313	2.409
	FDDP	PMDP	− 3.755	9.175	0.683	− 21.916	14.407	FDDP	PMDP	− 0.548	0.940	0.561	− 2.409	1.313
Female	PMDP	FDDP	33.410*	8.829	0.000	15.935	50.886	PMDP	FDDP	4.613*	0.893	0.000	2.846	6.381
	FDDP	PMDP	− 33.410*	8.829	0.000	− 50.886	− 15.935	FDDP	PMDP	− 4.613*	0.893	0.000	− 6.381	− 2.846

The dependent variables are CK and CK-MB.

(I) PMDP = 1, FDDP = 2; (J) PMDP = 1, FDDP = 2.

*P < 0.05.

## Discussion

In this study, serum CK levels and CK-MB levels of PMDP were significantly higher than those of FDDP after treatment with SSIRs, especially in females, indicating that repeated use of SSRIs may be related to an increase in serum CK and CK-MB levels.

CK is an important enzyme that catalyses the reversible phosphorylation of ATP and creatine to ADP and phosphocreatine in cellular energy metabolism^15,16^. CK is present as three isoenzymes: CK-MB (mostly in the heart), CK-MM (mostly in the muscle), or CK-BB (mostly in the brain)^17^. Elevated serum CK levels are more common in diseases with damaged muscle cell membranes, such as myocardial infarction (MI). CK-MB activity has been recognized as a specific and sensitive biomarker of clinical and subclinical myocardial injury^18,19^. A previous biopsy specimen study showed that normal myocardium contained only a small amount of CK-MB, while pressure overload myocardial hypertrophy and coronary artery disease increased CK-MB due to myocardial tissue hypoxia^20^. CK-MB levels are significantly positively correlated with the extent of myocardial injury, so serum CK-MB can be used as a surrogate marker for MI scope^21,22^. Elevation of these serum markers in this study did not exceed the normal upper limit, but it may indicate a tendency for long-term use to accumulate toxicity. In addition, a previous study demonstrated that a slight increase in CK-MB may be associated with several small areas of myocardial necrosis^18^, and even a slight increase in CK-MB indicated the possibility of coronary heart disease or myocardial infarction^23,24^.

There has been no previous report focusing on CK, CK-MB and SSRIs. In the present study, both CK and CK-MB levels were elevated after SSRI use and were related to the number of SSRI treatments.

Myocardial ischaemia might be the reason for the slight increase in CK-MB isozymes. Since serotonin was first isolated from serum (sero-) in the mid-twentieth century, it has been found to significantly promote smooth muscle contraction (-tonin). Stimulation of 5-HT_1B_, 5-HT_1D_ and 5-HT_2A_ receptors, causes coronary artery vasoconstriction, mediates myocardial ischaemia and increases CK-MB^25^. As an inhibitor of serotonin reuptake, the use of SSRIs has been reported to be associated with vasoconstriction and consequent myocardial ischaemia (Prinzmetal’s angina)^26,27^. However, the effects of SSRIs on the cardiovascular system are much more complex. Serotonin can cause either vasoconstriction or vasodilatation, and may increase or decrease blood pressure depending on the area of the vasculature^25,28^. Further studies are warranted to clarify these mechanisms. Another possible mechanism is that SSRIs inhibit extracardiac delayed rectifier potassium currents (IKr), which are important for cardiac repolarization. Inhibition of IKr may lead to severe arrhythmias and myocardial toxicity, especially in patients with CVD^29–31^. It is also possible that both mechanisms are in play simultaneously. TCAs are known to cause tachycardia and are associated with an increased risk for MI^32,33^.

A large prospective community study recently published by the American Heart Association (AHA) reported that SSRI use was not associated with a reduced risk of CVD compared to TCA use^34^, indicating that SSRIs are also associated with an increased risk of impaired heart function, leading to myocardial muscle damage and specific biomarker leakage into the blood through myocardial cell membranes. This finding is consistent with the cohort study of Blanchett et al., which suggested that in older people, SSRIs may increase the risk of acute myocardial infarction (AMI) compared to nonantidepressants, and that the risk may increase with the duration of antidepressant use^35^. Other indicators, such as cardiac baroreflex function, heart rate variability, pulse pressure and hsCRP, were also affected by SSRIs^36^. In contrast, some previous studies have observed that there is no association between SSRI use and coronary heart disease risk^37–39^. Some studies have even demonstrated that SSRIs are associated with protective effects regarding AMI. The hypothetical mechanism is that SSRIs reduce levels of platelet serotonin, weakening platelet activation and aggregation^40,41^. These inconsistencies might be due to differences in the characteristics of study participants, sample size, strategies for controlling depressive symptoms, and differences in specific results and follow-up time.

There is often a two-way relationship between MDD, immune metabolism, and CVD^42^. A bidirectional relationship between MDD and metabolic and cardiovascular diseases may underlie MDD and could be a precipitating and perpetuating factor for immunometabolic dysregulation, which is more often observed in MDD than in the healthy population^43–45^. MDD can promote the inflammatory response^46^, and the inflammatory process can promote the progression of CVD and the occurrence of adverse cardiac events^47^. The metabolic and immune system abnormalities observed in MDD patients may also be due to genetic polygenic effects that predispose these patients to the development of cardiovascular disease^48,49^. For instance, BDNF Val66Met is highly associated with the occurrence of depression^50^. Recently, it was found that BDNF Val66Met also increased the risk of AMI in humans by regulating blood coagulation and inflammation^51^. This evidence suggests that people with MDD are at higher risk for CVD.

It is worth noting that patients with depression have some characteristics that are considered to be cardiovascular risk factors, including age, race, gender, smoking, body mass index (BMI) and so on^52–55^. Haukala et al.^56^ showed that depressive symptoms predicted new CVD in female, whereas depressive symptoms did not predict new CVD in male. However, other study had noted that depression scores in male had been found to be associated with non-fatal coronary heart disease^57^. Our results revealed that CK and CK-MB were significantly higher in females female PMDP than in female FDDP, indicating that in females, there is a higher risk of myocardial muscle damage, but no relevant research among sex, antidepressants and CVDs exists at present. SSRIs have been found to affect the content of female sex hormones^58^, and repeated use of SSRIs may further affect female sex hormones, which may be one of the potential reasons for the higher levels of CK and CK-MB in female patients with PMDP than in FDDP. Moreover, there are sex differences in the metabolic pathways of patients with depression^59^, and female patients tend to have more adverse drug reactions^60^. Additional studies are needed to clarify the mechanism of antidepressant use and cardiovascular function in females.

This study has several limitations that should be addressed. First, this study lacked precise information on the prior dosage and treatment duration of PMDP. Second, since this is a retrospective study, we were unable to record or control for some factors that may affect cardiac function, such as cigarette smoking, BMI, blood pressure, and dietary habits. Future prospective studies should be designed to remedy this deficiency. Third, a previous study showed that SSRIs were associated with arrhythmias and a prolonged QTc interval of ECG. Unfortunately, we did not have complete ECG records for the subjects in this study, so we were unable to investigate the relationship between QT intervals, heart rate variability and SSRIs. Fourth, in this study, we measured the activity of CK-MB rather than the mass of CK-MB, which may lead to false elevation of CK-MB in macrocreatine kinases (type 1 or 2)^18^. However, macrocreatine kinase is a relatively rare condition. Fifth, there may be selection bias between medicated patients and nonmedicated patients, and the possibility that patients receiving medicated treatment may have more serious conditions cannot be ruled out. Finally, although our findings attempted to demonstrate that SSRIs might increase serum CK and CK-MB when reused, we lacked a time-toxicity relationship, as the sample size did not support this analysis.

## Conclusions

To the best of our knowledge, this is the first observational study to evaluate the effects of SSRIs on serum creatine kinase and creatine kinase-MB levels in patients with major depressive disorder. Our findings show that patients with MDD, especially the females, who have previously taken SSRI antidepressants may be at a higher risk of elevated CK and CK-MB, which are indicators of myocardial muscle damage. Further studies are needed to investigate the cardiotoxic effects of SSRIs and how to properly monitor CK and CK-MB in MDD patients with repeated use of SSRIs.
